# Antiplatelet therapy in aneurysmal subarachnoid hemorrhage: an updated meta-analysis

**DOI:** 10.1007/s10143-023-02120-2

**Published:** 2023-09-04

**Authors:** Keng Siang Lee, Cheyenne Lee, Permesh S. Dhillon, Ramez Kirollos, Vincent D.W. Nga, Tseng Tsai Yeo, Hans Henkes, Adam S. Arthur, Leonard L.L. Yeo, Pervinder Bhogal

**Affiliations:** 1 Department of Neurosurgery, King’s College Hospital, London, UK; 2 Department of Basic and Clinical Neurosciences, Maurice Wohl Clinical Neuroscience Institute, Institute of Psychiatry, Psychology and Neuroscience (IoPPN), King’s College London, London, UK; 3 Department of Psychological Medicine, King’s College London, Institute of Psychiatry, Psychology and Neuroscience (IoPPN), London, UK; 4 Interventional Neuroradiology, Queens Medical Centre, Nottingham University Hospitals NHS Trust, Nottingham, UK; 5 Department of Neurosurgery, National Neuroscience Institute, Singapore, Singapore; 6 Division of Neurosurgery, Department of Surgery, National University Health System, Singapore, Singapore; 7 Neuroradiologische Klinik, Neurozentrum, Klinikum Stuttgart, Stuttgart, Germany; 8 Medical Faculty, University Duisburg-Essen, Essen, Germany; 9 Department of Neurosurgery, Semmes-Murphey Clinic, University of Tennessee Health Science Center, Memphis, TN USA; 10 Division of Neurology, Department of Medicine, National University Health System, Singapore and Yong Loo Lin School of Medicine, National University of Singapore, Singapore, Singapore; 11 Department of Interventional Neuroradiology, The Royal London Hospital, Barts NHS Trust, London, UK

**Keywords:** Aneurysm, Antiplatelet, Ischemia, Neuroprotection, Stroke, Subarachnoid hemorrhage, Vasospasm, Meta-analysis

## Abstract

Antiplatelet therapy (AT) may serve to reduce the effects of aneurysmal subarachnoid hemorrhage (aSAH)-induced pro-coagulant state in the cerebral circulation. Several studies, however, have delivered conflicting conclusions on the efficacy of AT post aSAH. Systematic searches of Medline, Embase, and Cochrane Central were undertaken on 27th March 2023. The primary outcome was delayed cerebral ischaemia (DCI). Secondary outcomes were symptomatic and angiographic vasospasm, good functional outcome (modified Rankin Scale [mRS] with scores 0–2), hemorrhagic events, and in-hospital mortality. Twenty-two studies reporting 4378 patients with aSAH were included in the meta-analysis. AT was associated with lower rates of DCI (*RR*=0.62, 95% CI: 0.43; 0.89), symptomatic vasospasm (*RR*=0.63, 95% CI: 0.46; 0.86), and moderate/severe angiographic vasospasm (*RR*=0.74, 95% CI: 0.65; 0.84), with no effect on hemorrhagic complications (*RR*=1.36, 95% CI: 0.77; 2.41). When analyzing only post-ictal use of AT, AT additionally favored rates of good functional outcomes (*RR*=1.18, 95% CI: 1.10; 1.26) and in-hospital mortality (*RR*=0.56, 95% CI: 0.39; 0.80). In the subgroup treated with cilostazol, AT was associated with lower rates of DCI (*RR*=0.40, 95% CI: 0.32), symptomatic vasospasm (*RR*=0.47, 95% CI: 0.33; 0.65), moderate/severe angiographic vasospasm (*RR*=0.75, 95% CI: 0.57; 0.98) and good functional outcome (*RR*=1.24, 95% CI: 1.08; 1.43). In the surgically treated aSAH subgroup, AT favored rates of symptomatic vasospasm (*RR*=0.55, 95% CI: 0.30; 0.98), moderate/severe angiographic vasospasm (*RR*=0.70, 95% CI: 0.54; 0.90) and good functional outcome (*RR*=1.23, 95% CI: 1.09; 1.41). In the endovascularly treated aSAH subgroup, AT was associated with lower rates of in-hospital mortality (*RR*=0.60, 95% CI: 0.41; 0.88). In aSAH patients, post-ictal AT is associated with benefits in terms of rates of DCI, vasospasm, good functional outcomes, and in-hospital mortality without an increased risk of hemorrhagic events.

**Supplementary Information:**

The online version contains supplementary material available at 10.1007/s10143-023-02120-2.

## Introduction

Delayed cerebral ischemia (DCI) occurs in approximately 30% aneurysmal subarachnoid hemorrhage (aSAH) patients and is associated with significant morbidity and mortality [1, 2]. Early recognition and prompt treatment of post aSAH cerebral vasospasm with cardiac output optimization are the mainstays of DCI prophylactic strategies [3, 4]. However, it was recently shown that induced hypertension was associated with increased rates of complications [5].

Although vasospasm with reduced cerebral blood flow and subsequent DCI were historically considered the cause of delayed neurological deterioration after aSAH, the pathogenesis of DCI remains to be fully elucidated. The absence of a convincing association between large vessel vasospasm and DCI has led to a search for alternative etiologies of DCI [6]. Several mechanisms have been proposed, which encompass endothelial dysfunction, inflammatory activation, microcirculatory dysfunction with loss of autoregulation, cortical spreading depolarization, and microthrombosis [7]. Alongside the above, an increased platelet aggregation and related release of thromboxane B2 post aSAH, create a prothrombotic environment in the cerebral vasculature with vasoconstriction, which have been suggested to lead to the development of DCI [8–11]. There is an ongoing pursuit to identify novel therapies for DCI post aSAH. Antiplatelet therapy (AT) may serve to reduce the effects of an aSAH-induced pro-coagulant state, thereby limiting microthrombotic and microembolic events in the cerebral circulation. Several studies have delivered conflicting conclusions on the efficacy of AT post aSAH; some have shown that AT can ameliorate the risk of DCI [12, 13], while others report no benefit. No consensus has been reached.

To arrive at an optimal clinical management protocol, this study aims to investigate the efficacy and safety of AT in patients with aSAH, through a systematic review and meta-analysis of the current literature. In addition, we explored the effect of the timing of AT and various AT types in relation to the treatment modality (i.e., surgical or endovascular treatment).

## Methods

The review was conducted according to the Preferred Reporting Items for Systematic Reviews and Meta-Analyses (PRISMA) guidelines [14]. The protocol was registered on the PROSPERO international prospective register of systematic reviews (registration number CRD42023 413704).

### Outcomes

The primary outcome was DCI. The definition of DCI among the studies was heterogenous but in general based on radiological and clinical criteria as defined by Vergouwen et al. [15]. Radiological DCI was defined as the presence of cerebral infarction on CT (new hypodensities) or MR (or new diffusion-restricted areas) scan of the brain within 6 weeks after SAH, or on the latest CT or MR scan made before death within 6 weeks, or proven at autopsy, and not attributable to other causes. Clinical deterioration caused by DCI was defined by the occurrence of focal neurological impairment (such as hemiparesis, aphasia, apraxia, hemianopia, or neglect), or a decrease of at least 2 points on the Glasgow Coma Scale, which cannot be attributed to other causes by means of clinical assessment, CT or MRI scanning of the brain, and appropriate laboratory studies.

Secondary outcomes were symptomatic and angiographic vasospasm, good functional outcome, any hemorrhagic events (intracranial and extracranial), and in-hospital mortality. Clinical vasospasm was defined as a significant decline in neurological examination findings, accompanied by impaired flow changes noted on CT perfusion study or radiological evidence on diagnostic cerebral catheter angiography. The degree of angiographic vasospasm was rated as “mild” when narrowing of the arterial diameter was <30%, “moderate” when 30 to 49%, and “severe” when ≥50%, with the vessel diameter on the initial angiography used as a reference. Good functional outcome was defined on the modified Rankin Scale (mRS) with scores of 0–2, at last follow-up. In studies where other outcome measures were reported, these outcomes were used if they were translatable into a good or poor outcome as defined above.

### Search strategy

Searches of the following three electronic databases were undertaken: Ovid Medline, Ovid Embase, and Cochrane Central Register of Controlled Trials (CENTRAL). Searches were performed in each database from its inception until 27 March 2023. The concepts of “subarachnoid hemorrhage,” “delayed cerebral ischemia,” “vasospasm,” and “antiplatelet” were used in addition to synonyms and related terms. The full search strategy used for the databases is presented in Supplementary Table 1.

### Study selection

All titles and abstracts were screened against the pre-defined eligibility criteria developed independently by two reviewers (KSL and CL). A full list of inclusion and exclusion criteria can be found in Supplementary Table 2. Disagreements were resolved by discussion, and where agreement could not be reached, the senior reviewer assisted with decision making (PB). Agreement among the reviewers on study inclusion was evaluated using Cohen’s kappa statistic [16].

In the event of multiple publications analyzing the same cohort, the publication that reported the largest patient data was used for evaluation. This was to avoid multiple counting which overstates sample size, leading to an artificially exaggerated precision in the pooled estimate [17]. The reference lists of included studies were also scrutinized to identify relevant studies fitting the inclusion criteria that may have been inadvertently overlooked in our search strategy [18].

### Data extraction

A pro forma was developed and piloted to extract data on the following variables to ensure standardization and consistency in this process: (1) study details, (2) study design, (3) participant demographics (Fisher grades, Hunt and Hess grades, World Federation of Neurosurgical Societies [WFNS] grades, aneurysm location), (4) country and dataset, (5) selection criteria, (6) type of antiplatelet and control, (7) indication for treatment, (8) results (DCI, vasospasm, functional outcome, in-hospital mortality, and complications).

### Risk of bias assessment

The quality of included studies was assessed using the Joanna Briggs Institute (JBI) checklist for non-randomized experimental studies and version 2 of the Cochrane risk-of-bias assessment for randomized trials (RoB 2) for randomized controlled trials (RCTs).

### Statistical analysis

Meta-analyses were performed assuming the random effects model to account for heterogeneity within and between individual studies [16].

To obtain risk ratios (RRs) from reported binary outcomes, pairwise meta-analysis was conducted using the Mantel–Haenszel method without continuity correction, using the Paule-Mandel estimator. Overall pooled proportions of demographic comorbidities of included patients were computed using the generalized linear mixed model (GLMM) method using a random intercept logistic regression model via logit transformation [16, 19]. Knapp–Hartung adjustments were used to reduce the chance of false positive and to control the estimate uncertainties of the between-study heterogeneity. GLMM instead of Freeman–Tukey double arcsine transformation was employed as GLMM has been shown to provide the most accurate estimate for meta-analysis of single proportions in simulation studies [16, 19].

The I^2^ statistic was used to present inter-study heterogeneity, where I^2^ ≤ 30%, between 30 and 50%, between 50 and 75%, and ≥ 75% were considered to indicate low, moderate, substantial, and considerable heterogeneity, respectively [20]. *P* values for the I^2^ statistic were derived from the chi-squared distribution of Cochran’s Q test. Prediction intervals were reported for all outcome measures. A prediction interval provides estimates of what the effect size might be for similar studies conducted in the future.

For pooling of means of numerical variables, we computed missing means and standard deviations (SDs) from medians, ranges (minimum to maximum) and interquartile ranges (IQRs) using the methods proposed by Hozo et al. and Wan et al. [21, 22].

In order to delineate the individual effects of the timing of antiplatelet administration, individual antiplatelet agents and treatment modality, subgroup analyses were performed for post-ictal and pre-ictal AT, various AT, surgically treated ruptured aneurysms, and endovascularly treated ruptured aneurysms. Importantly, the controls defined in our study were limited to only patients not administered AT post SAH in our post-ictal analysis, and patients previously unexposed to AT prior to SAH, in our pre-ictal analysis.

Summary-level meta-regression was performed using mixed-effect meta-analysis model by GLMM method, to identify predictors of DCI including older age, higher clinical and radiographic grades of aSAH, and acute hydrocephalus, in accordance with literature [7].

Publication bias of studies was assessed using funnel plots, where an asymmetrical distribution of studies was suggestive of bias. Quantitative analysis of funnel plot asymmetry was done using Egger’s regression test. The GRADE approach was used to evaluate the quality of evidence for each outcome [23].

All statistical analyses were performed using R software version 4.2.1 (R Foundation for Statistical Computing, 2022), with the package *meta. P*-values less than 0.05 were considered statistically significant.

## Results

### Overview of included studies

The systematic search yielded 1726 unique publications. After screening of titles and abstracts, 54 publications were reviewed in full text. A total of 22 studies, of 4378 patients with aSAH met the eligibility criteria for inclusion in our meta-analysis (Supplementary Figure 1) [8, 12, 13, 24–40]. The study by Shimamura et al. was excluded from analysis as AT use occurred pre-ictal which continued during procedures [41]. Reliability of study selection between observers was substantial at both the title and abstract screening stage (Cohen’s *κ*=0.92) and the full-text review stage (Cohen’s *κ*=1.00) [42].

Eight randomized controlled trials and 14 non-randomized cohort studies were included. Data were collected across seven countries — one from Finland, three from Germany, 10 from Japan, one from Korea, two from the Netherlands, one from Switzerland, and four from the USA. Details of included studies, including types and doses of AT administered, are reported in Supplementary Table 3. On assessing the risk of bias using the JBI checklist, 12 studies attained a full score of 11, while one each attained a score of 10 and 9 (Supplementary Table 4). On assessing the risk of bias using the RoB-2 checklist, four RCTs had low risk of bias, two had some concerns, and two had high risk of bias (see Supplementary Table 4, 5).

### Patient baseline characteristics and workflow

Of the 4378 patients, 1645 were treated with AT, whereas 2733 patients did not receive antiplatelet agents. The antiplatelet agent was cilostazol in seven studies, thromboxane A2 synthetase inhibitors (OKY-046) in three, aspirin monotherapy in seven, and aspirin with clopidogrel in five. Post-ictal AT administration ranged between one to greater than 6 weeks, with most regimens lasting 2 weeks.

Gender of the patients was reported in 19 of 22 studies — 28.1% and 32.7% were male in the AT and non-AT groups, respectively. The mean and SD of their age were reported or imputable in 16 of 22 studies. Overall pooled mean age across the AT and non-AT groups were 57.0 years (95% CI: 54.1; 60.0, *I*^2^=93.6% [*p*<0.001]) and 56.8 (95% CI: 53.5; 60.1, *I*^2^=94.9% [*p*<0.001]), respectively. Type of treatment modality of aneurysms was reported in 21 of 22 studies — 52.0% and 53.0% were treated by microsurgical clipping in the AT and non-AT groups, respectively.

Pooled prevalence of baseline characteristics, including SAH grades, stratified according to treatment arm, is summarized in Table 1. There were no baseline differences between the groups. Clinical follow-up ranged from discharge to 1 year.

**Table 1:** Pooled baseline characteristics of included patients

Characteristic	Antiplatelet				No antiplatelet			
	No. of studies	Pooled effect size (95% CI)	I^2^ (%)	*P* value of I^2^ (from χ^2^ test)	No. of studies	Pooled effect size (95% CI)	I^2^ (%)	*P* value of I^2^ (from χ^2^ test)
Mean age (SD), year	16	57.0 [54.1; 60.0]	93.6	<0.001	16	56.8 [53.5; 60.1]	94.9	<0.001
Hypertension	6	47.00 [36.20; 58.08]	67.9	0.008	6	38.71 [29.80; 48.45]	82.9	<0.001
Diabetes mellitus	3	9.96 [4.20; 21.82]	0.0	0.405	3	5.72 [0.79; 31.61]	86.3	<0.001
Fisher 3 and 4	12	80.63 [70.53; 87.86]	82.5	<0.001	12	83.63 74.52; 89.93]	90.0	<0.001
Hunt and Hess III and IV	10	44.49 [35.65; 53.69]	64.6	0.003	10	52.16 [38.16; 65.83]	84.8	<0.001
WFNS IV and V	9	32.35 [26.52; 38.78]	55.1	0.023	9	32.63 [24.37; 42.14]	79.4	<0.001
MCA	13	25.33 [18.50; 33.64]	63.8	<0.001	13	18.97 [14.17; 24.92]	58.6	0.004
ICA	14	25.00 [22.04; 28.22]	0.0	0.665	14	24.65 [16.97; 34.37]	79.5	<0.001
Vertebrobasilar	11	7.96 [3.60; 16.67]	88.4	<0.001	11	10.52 [4.82; 21.43]	91.5	<0.001
Acute hydrocephalus	9	49.04 [29.51; 68.86]	89.3	<0.001	9	54.45 [36.91; 70.96]	89.3	<0.001

### Primary outcome — delayed cerebral ischemia

**Fig. 1:**
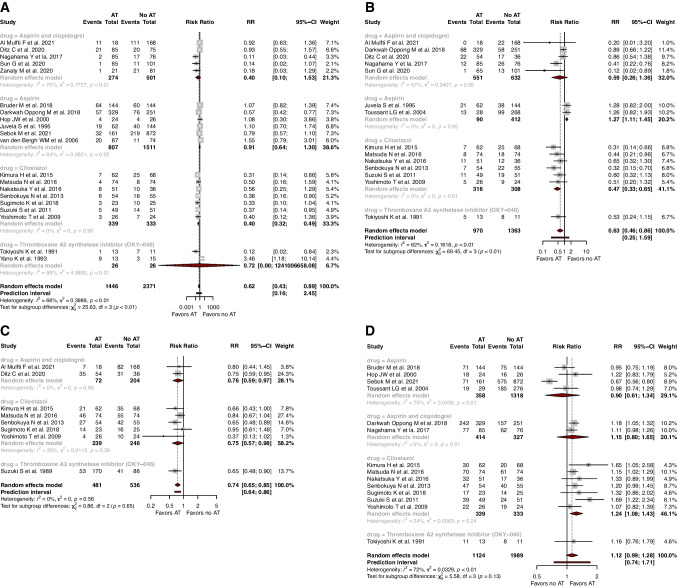
Forest plots with random-effects model, stratified by individual antiplatelets, of **A** delayed cerebral ischemia, **B** symptomatic vasospasm, **C** moderate/severe angiographic vasospasm, and **D** good functional outcome (mRS0-2)

DCI was reported across 20 studies, including 3817 patients. Compared with the non-AT group, lower rates of DCI were reported in the AT group (*RR*=0.62, 95% CI: 0.43; 0.89, prediction interval: 0.16; 2.45, *I*^2^=66%, *p*<0.001) (Fig. 1). On meta-regression, age (*p*=0.662) and proportions of Fisher grade 3 and 4 (*p*=0.928), Hunt and Hess III and IV (*p*=0.340), WFNS IV and V (*p*=0.707), posterior circulation aneurysms (*p*=0.738), and acute hydrocephalus (*p*=0.324) were not statistically significant predictors of DCI post aSAH.

### Secondary outcomes

Compared with non-AT, AT was associated with lower rates of symptomatic vasospasm (*RR*=0.63, 95% CI: 0.46; 0.86, prediction interval: 0.25; 1.59, *I*^2^=62%, *p*=0.001), moderate/severe angiographic vasospasm (*RR*=0.74, 95% CI: 0.65; 0.84, prediction interval: 0.64; 0.86, *I*^2^=0, *p*=0.550) and severe angiographic vasospasm (*RR*=0.66, 95% CI: 0.51; 0.84, prediction interval: 0.50; 0.87, *I*^2^=71.7, *p*<0.001).

**Fig. 2:**
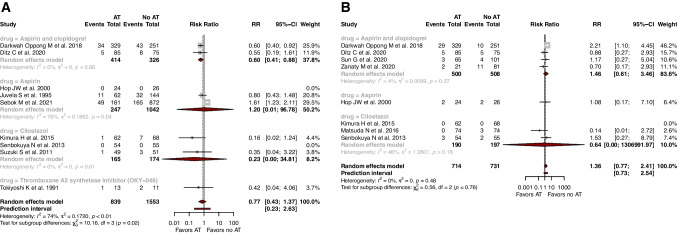
Forest plots with random-effects model, stratified by individual antiplatelets, of **A** in-hospital mortality, and **B** hemorrhagic complications

The effect of AT on rates of good functional outcome (*RR*=1.12, 95% CI: 0.99; 1.28, prediction interval: 0.74; 1.71, *I*^2^=72%, *p*<0.001), in-hospital mortality (*RR*=0.77, 95% CI: 0.43; 1.37, prediction interval: 0.23; 2.63, *I*^2^=74%, *p*<0.001), and hemorrhagic complications (*RR*=1.36 95% CI: 0.77; 2.41, prediction interval: 0.73; 2.54, *I*^2^=0, *p*=0.476) was not significant (Fig. 2) (Table 2).

**Table 2:** Pooled outcomes of included patients

Outcome	No. of studies reporting variable	No. of patients analyzed	Pooled effect size (95% confidence interval) [prediction interval]	I^2^ (%)	*P* value of I^2^ (from χ^2^ test)	Quality of evidence (GRADE)
Delayed cerebral ischemia	20	3817	RR0.62 (0.43; 0.89) [0.16; 2.45]	65.8	<0.001	Low
Symptomatic vasospasm	14	2333	RR0.63 (0.46; 0.86) [0.25; 1.59]	62.1	0.001	Low
Angiographic vasospasm (moderate and severe)	8	1017	RR0.74 (0.65; 0.84) [0.64; 0.86]	0.0	0.550	Low
Angiographic vasospasm (severe)	7	989	RR0.66 (0.51; 0.84) [0.50; 0.87]	0.0	0.546	Low
mRS score 0–2	14	3113	RR1.12 (0.99; 1.28) [0.74; 1.71]	71.7	<0.001	Low
In-hospital mortality	7	2392	RR0.77 (0.43; 1.37) [0.23; 2.63]	74.1	<0.001	Low
Hemorrhagic complications	7	1445	RR1.36 (0.77; 2.41) [0.73; 2.54]	0.0	0.476	Low

*mRS* modified Rankin Scale. No antiplatelet group used as control

### Subgroup analyses by timing of antiplatelet administered

Only outcomes with more than one included study in the subgroup analysis are reported (Supplementary Table 6).

**Fig. 3:**
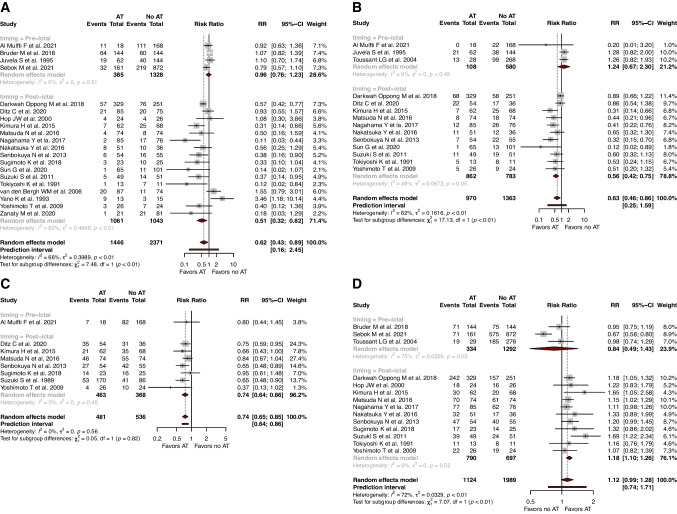
Forest plots with random-effects model, stratified by timing of antiplatelet use, of **A** delayed cerebral ischemia, **B** symptomatic vasospasm, **C** moderate/severe angiographic vasospasm, and **D** good functional outcome (mRS0-2)

**Fig. 4:**
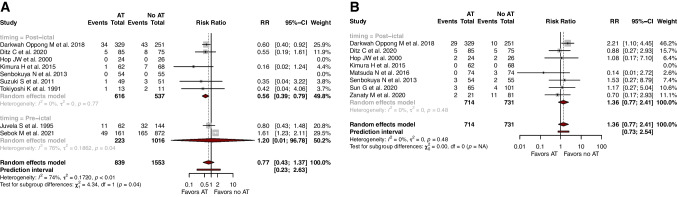
Forest plots with random-effects model, stratified by timing of antiplatelet use, of **A** in-hospital mortality, and **B** hemorrhagic complications

When specifically looking at post-ictal use of AT, AT was associated with improved rates of DCI (*RR*=0.50, 95% CI: 0.32; 0.82, *I*^2^=0), symptomatic vasospasm (*RR*=0.56, 95% CI: 0.42; 0.75, *I*^2^=0), and moderate/severe angiographic vasospasm (*RR*=0.74, 95% CI: 0.64; 0.86, *I*^2^=0) (Fig. 3). In addition, the use of AT was associated with increased rates of good functional outcomes (*RR*=1.18, 95% CI: 1.10; 1.26 *I*^2^=0) and lowered rates of in-hospital mortality (*RR*=0.56, 95% CI: 0.39; 0.80, *I*^2^=0) (Fig. 4).

When specifically looking at pre-ictal use of AT, AT was not associated with any benefits, in terms of rates of DCI (*RR*=0.96, 95% CI: 0.76; 1.23, *I*^2^=62.3), symptomatic vasospasm (*RR*=1.24, 95% CI: 0.67; 2.30, *I*^2^=0), good functional outcomes (*RR*=0.84, 95% CI: 0.49; 1.43, *I*^2^=74.7), and in-hospital mortality (*RR*=1.20, 95% CI: 0.01; 96.78, *I*^2^=75.9).

### Subgroup analyses by type of antiplatelet administered

In the subgroup treated with cilostazol, AT was associated with lower rates of DCI (*RR*=0.40, 95% CI: 0.32; 0.49, *I*^2^=0), symptomatic vasospasm (*RR*=0.47, 95% CI: 0.33; 0.65, *I*^2^=0), moderate/severe angiographic vasospasm (*RR*=0.75, 95% CI: 0.57; 0.98, *I*^2^=20.3), and good functional outcome (*RR* =1.24, 95% CI: 1.08; 1.43, *I*^2^=24). The effect of AT on rates of severe angiographic vasospasm (*RR*=0.59, 95% CI: 0.28; 1.27, *I*^2^=28), in-hospital mortality (*RR*=0.23, 95% CI: 0.00; 34.81, *I*^2^=0), and hemorrhagic complications (*RR*=0.64, 95% CI: 0.00; 1306991.97, *I*^2^=46) was not significant (Supplementary Table 7).

In the subgroup treated with dual therapy of aspirin and clopidogrel, AT favored rates of moderate/severe angiographic vasospasm (*RR*=0.76, 95% CI: 0.59; 0.97, *I*^2^=0) and in-hospital mortality (*RR*=0.60, 95% CI: 0.41; 0.88, *I*^2^=0). The effect of AT on rates of DCI (*RR*=0.40, 95% CI: 0.10; 1.53, *I*^2^=71), symptomatic vasospasm (*RR*=0.59, 95% CI: 0.26; 1.36, *I*^2^=57), good function outcome (*RR*=1.15, 95% CI: 0.80; 1.65, *I*^2^=0), and hemorrhagic complications (*RR*=1.46, 95% CI: 0.61; 3.46, *I*^2^=4.3) was not significant.

In the subgroup treated with monotherapy of aspirin, AT was associated with greater rates of symptomatic vasospasm (*RR*=1.27, 95% CI: 1.11; 1.45, *I*^2^=0). It is to be noted that the two studies included for this outcome in aspirin monotherapy, however, were pre-ictal uses of AT (Fig. 1) [37, 43]. The effect of AT on rates of DCI (*RR*=0.91, 95% CI: 0.64; 1.30, *I*^2^=64), good functional outcome (*RR*=0.90, 95% CI: 0.61; 1.34, *I*^2^=75), and in-hospital mortality (*RR*=1.20, 95% CI: 0.01; 3.47, *I*^2^=76) was not significant.

### Subgroup analyses by treatment modality

In the surgically treated aSAH subgroup, AT was associated with lower rates of symptomatic vasospasm (*RR*=0.55, 95% CI: 0.30; 0.98, *I*^2^=71), moderate/severe angiographic vasospasm (*RR*=0.70, 95% CI: 0.54; 0.90, *I*^2^=0), and good functional outcome (*RR*=1.23, 95% CI: 1.09; 1.41, *I*^2^=13) (Supplementary Figure 2). The effect of AT on rates of DCI (*RR*=0.58, 95% CI: 0.26; 1.31, *I*^2^=73), severe angiographic vasospasm (*RR*=0.61, 95% CI: 0.21; 1.81, *I*^2^=24), in-hospital mortality (*RR*=0.65, 95% CI: 0.28; 1.52, *I*^2^=0), and hemorrhagic complications (*RR*=1.30, 95% CI: 0.15; 11.50, *I*^2^=0) was not significant in the surgically treated aSAH subgroup.

In the endovascularly treated aSAH subgroup, AT was associated with lower rates of in-hospital mortality (*RR*=0.60, 95% CI: 0.41; 0.88, *I*^2^=0). The effect of AT on rates of DCI (*RR*=0.37, 95% CI: 0.11; 1.21, *I*^2^=66), symptomatic vasospasm (*RR*=0.60, 95% CI: 0.20; 1.80, *I*^2^=65), good functional outcome (*RR*=1.15, 95% CI: 0.80; 1.65, *I*^2^=0), and hemorrhagic complications (*RR*=1.46, 95% CI: 0.61; 3.46, *I*^2^=4) was not significant in the endovascularly treated aSAH subgroup (Supplementary Table 8). A sensitivity analysis of only post-ictal studies conferred the same results suggesting robust findings (Supplementary Table 9).

## Discussion

### Summary of findings

This updated meta-analysis including data on 4378 patients with aSAH found that compared with non-AT treatment, AT was associated with reduced occurrence of DCI and both symptomatic and angiographic vasospasm, with no increased risk of hemorrhagic complications. Specifically in the post-ictal AT group, these benefits translated to improved functional outcomes and reduced in-hospital mortality rates. In the subgroup analyses for each individual antiplatelet agent, outcomes of cilostazol treatment echoed those of the overall analysis. Dual therapy of aspirin and clopidogrel conferred benefits in terms of rates of angiographic vasospasm, good functional outcomes, and in-hospital mortality. The lack of benefit to DCI in this subgroup is likely due to a type 2 error. These benefits were not replicated in the aspirin monotherapy cohort. Subgroup analysis by treatment modality revealed that surgically treated aSAH was associated with lower rates of symptomatic and angiographic vasospasm and greater rates of good functional outcomes, in the AT group. Another subgroup analysis of endovascularly treated aSAH found a lower rate of in-hospital mortality in the AT cohort. Overall, our findings suggest that the use of AT in aSAH is safe and may confer neuroprotection.

### Comparison with literature

Data within the literature about the influence of AT on the occurrence of DCI after aSAH are heterogeneous and contradictory [44–47]. Close to two decades ago, Mees et al. found a trend towards improved functional outcomes in patients with aSAH treated with AT, possibly due to a reduction in DCI. However, these results were not statistically significant, thus no definite conclusions could be drawn [47]. Studies included in those older reviews reported mainly surgically treated patients, which is not as representative of the modern treatment management of aSAH, especially after results of the International Subarachnoid Aneurysm Trial (ISAT) which suggested that in patients with aSAH suitable for both clipping and endovascular treatment, those with endovascular treatment were more likely to survive independently [48]. In our study, only half of the patients underwent clipping which is closer to modern reflection of the neurovascular practice. It has been demonstrated that clipping is associated with increased risk of DCI compared with coiling [49, 50]. Manipulation of the brain and vessel wall during surgery is purported to drive vasospasm and DCI [49, 50]. In addition, patients were administered mainly aspirin monotherapy which might not have been the optimal antiplatelet [38, 46, 47]. However, our secondary analysis on the surgically treated cohort showed AT was associated with lower rates of symptomatic and angiographic vasospasm, which corroborates those of Snyder and colleagues, although they had found the added benefit of functional independence in this specific cohort of patients [45]. Although endovascular treatment avoids the complications of neurosurgical clipping, it is associated with thromboembolic events. Thrombotic events after aSAH include cerebral infarction and microthrombus, both of which are included in the definition of DCI. It is considered standard practice during endovascular procedures to use heparin and monitor the activated clotting time in order to minimize any thromboembolic complications. With the advent of dedicated neurovascular stents, neck bridging devices, and flow diversion, there has been a greater use of these devices in the acute setting. The use of such devices necessitates the appropriate use anti-platelets and typically early on this revolved around the use of aspirin and clopidogrel. More recently, however, there has been a shift towards newer P2Y12 agents such as ticagrelor and prasugrel given the high non-response rate for clopidogrel [51]. Our subgroup analysis of endovascularly treated aSAH showed AT conferred lower rates of in-hospital mortality in the AT cohort; however, these findings were not consistent in the dual antiplatelet cohort, and this could be due to the fact that a large number of patients are clopidogrel non-responders.

More recent meta-analyses with DCI as their primary outcome report different effect estimates. The study by Cagnazzo et al., overall, failed to show a significant beneficial effect of AT on the occurrence of DCI and vasospasm; however, it conferred better functional outcomes and lower rates of mortality [44]. In the subgroup of patients with endovascular treatment, AT tended to be associated with a reduction of DCI. Despite the overall reduction of DCI not reaching statistical significance, neurologic functional outcome in patients who received platelet inhibitors was significantly better, and mortality was significantly lower. A caveat of their study involves including a study with pre-ictal aspirin use [43] and multiple counting from the same MASH and ISAT studies, which could have exaggerated the precision of the estimate in the wrong direction [38, 52, 53]. Our findings in post-ictal use of AT hence do not support those of Cagnazzo et al. as AT was indeed associated with reduced occurrence of DCI and both symptomatic and angiographic vasospasm in patients with aSAH, which also translated to improved functional outcomes and reduced in-hospital mortality rates. Our findings are similar to that of Snyder et al. [45] who focused only on the use of AT after aSAH, although had included a pre-ictal study [41]. These findings were also replicated in the subgroup analysis of cilostazol monotherapy. Notably, our subgroup analyses revealed that cilostazol monotherapy contributed most to the significant effect estimates of AT found in the primary analysis. Cilostazol is a unique antiplatelet agent that has been commercially available and can be readily repurposed for aSAH [54]. As a phosphodiesterase III inhibitor, it reversibly inhibits platelet aggregation and additionally possesses vasodilatory and antiproliferative properties on smooth muscles [55, 56]. The therapeutic response of cilostazol on DCI may hence be due to its multiple effects, in tandem, on the various pathways involved in DCI [55, 56].

### Limitations

Limitations of our meta-analysis are a result of including retrospective and observational studies with notable heterogeneity between them. There was no standard time frame with different lengths of clinical follow-up in each study. In an effort to address inherent heterogeneity, subgroup analyses were performed, which allowed us to delineate the effect of individual antiplatelets and treatment modality. However, most of the ruptured aneurysms in our study were treated with cilostazol, so our subgroup analysis by types of AT in aSAH was limited by a small number of studies with considerable missing data. The influence of the dose of various AT could not be investigated because the available data did not allow further subgroup analysis. The lack of significant findings within the subgroups may be a function of a type 2 error, however. Finally, the majority of included studies were performed in Japan, and hence, the results of our findings could have been partly confounded by differences in genetic and environmental risk factors, limiting its external validity. Studies in non-Japanese populations are therefore warranted to further evaluate the role of AT, in particular cilostazol, as a potential neuroprotective agent for aSAH. Nonetheless, this updated work is the largest to date analyzing the use of AT among patients with aSAH, and its strength includes avoiding undue emphasis on individual studies, thus yielding risk estimates that are more reliable.

## Conclusion

This updated meta-analysis reveals that in aSAH patients, post-ictal AT is associated with benefits in terms of rates of DCI, vasospasm, good functional outcomes, and in-hospital mortality without an increased risk of hemorrhagic events. Specifically, cilostazol monotherapy, an inhibitor of platelet aggregation, and cerebral vasodilator, contributed most consistently to the observed effect size. Future RCTs are needed to validate the role of AT.

### Supplementary information

ESM 1 (DOCX 1221 kb)

## Data Availability

Supplementary file.
