# Supplementary material for: Antiplatelet therapy in aneurysmal subarachnoid hemorrhage: an updated meta-analysis
Source: Neurosurg Rev. 2023 Sep 4;46(1):221. doi: 10.1007/s10143-023-02120-2 (PMC10477151; doi:10.1007/s10143-023-02120-2)
Supplement: Supplementary file 1 — (DOCX 1221 kb) [file 10143_2023_2120_MOESM1_ESM.docx]

Supplementary Table 1. Search strategy used for the three electronic databases 27 March 2023

| **EMBASE search** | | **1667 articles** | |
| --- | --- | --- | --- |
| No. | Search term | | |
| **Subarachnoid hemorrhage concept** | | | |
| 1 | exp subarachnoid hemorrhage/ | | |
| 2 | (subarachnoid haemorrhage or subarachnoid hemorrhage).tw. | | |
| 3 | 1 or 2 | | |
| **Delayed cerebral ischemia concept** | | | |
| 4 | exp brain ischemia/ or exp brain vasospasm/ or exp vasospasm/ | | |
| 5 | (delayed cerebral ischemia or delayed cerebral ischaemia).tw. | | |
| 6 | vasospasm.tw | | |
| 7 | 4 or 5 or 6 | | |
| **Antiplatelet concept** | | | |
| 8 | exp dual antiplatelet therapy/ or exp antiplatelet activity/ or exp acetylsalicylic acid/ or exp cilostazol/ or exp phosphodiesterase inhibitor/ or exp purinergic P2Y12 receptor/ or exp antithrombocytic agent/ | | |
| 9 | antiplatelet.tw or aspirin.tw or acetylsalicylic acid.tw or cilostazol.tw or clopidogrel.tw or P2Y12.tw or GP2b3a.tw or phosphodiesterase.tw | | |
| 10 | 8 or 9 | | |
| **Combined concepts** | | | |
| 11 | 3 and 7 and 10 | | |
| **OVID Medline search** | | | **305 articles** |
| No. | Search term | | |
| **Subarachnoid hemorrhage concept** | | | |
| 1 | exp Subarachnoid Hemorrhage/ | | |
| 2 | (subarachnoid haemorrhage or subarachnoid hemorrhage).tw. | | |
| 3 | 1 or 2 | | |
| **Delayed cerebral ischemia concept** | | | |
| 4 | exp brain ischemia/ or exp ischemia/ or exp Vasospasm, Intracranial/ | | |
| 5 | (delayed cerebral ischemia or delayed cerebral ischaemia).tw. | | |
| 6 | vasospasm.tw | | |
| 7 | 4 or 5 or 6 | | |
| **Antiplatelet concept** | | | |
| 8 | exp Platelet Aggregation Inhibitors/ or exp Aspirin/ or exp Cilostazol/ or exp Receptors, Purinergic P2Y12/ or exp Purinergic P2Y Receptor Antagonists/ | | |
| 9 | (antiplatelet or aspirin or acetylsalicylic acid or cilostazol or clopidogrel or P2Y12 or GP2b3a or phosphodiesterase).tw | | |
| 10 | 8 or 9 | | |
| **Combined concepts** | | | |
| 11 | 3 and 7 and 10 | | |
| **Cochrane Central Register of Controlled Trials (CENTRAL**) | | **52 articles** | |
| No. | Search term | | |
| **Subarachnoid hemorrhage concept** | | | |
| 1 | MeSH descriptor: [Subarachnoid Hemorrhage] explode all trees | | |
| 2 | (subarachnoid haemorrhage or subarachnoid hemorrhage):ti,ab,kw | | |
| 3 | #1 or #2 | | |
| **Delayed cerebral ischemia concept** | | | |
| 4 | MeSH descriptor: [Brain Ischemia] explode all trees | | |
| 5 | MeSH descriptor: [Ischemia] explode all trees | | |
| 6 | MeSH descriptor: [Vasospasm, Intracranial] explode all trees | | |
| 7 | (delayed cerebral ischemia or delayed cerebral ischaemia or vasospasm):ti,ab,kw | | |
| 8 | #4 or #5 or #6 or #7 | | |
| **Antiplatelet concept** | | | |
| 9 | MeSH descriptor: [Platelet Aggregation Inhibitors] explode all trees | | |
| 10 | MeSH descriptor: [Aspirin] explode all trees | | |
| 11 | MeSH descriptor: [Cilostazol] explode all trees | | |
| 12 | MeSH descriptor: [Receptors, Purinergic P2Y12] explode all trees | | |
| 13 | MeSH descriptor: [Purinergic P2Y Receptor Antagonists] explode all trees | | |
| 14 | (antiplatelet or aspirin or acetylsalicylic acid or cilostazol or clopidogrel or P2Y12 or GP2b3a or phosphodiesterase):ti,ab,kw | | |
| 15 | #9 or #10 or #11 or #12 or #12 or #13 or #14 | | |
| **Combined concepts** | | | |
| 16 | #3 and #8 and #15 | | |

Supplementary Table 2. Inclusion and exclusion criteria used to select studies for the review

| Inclusion criteria | Exclusion criteria |
| --- | --- |
| Primary interventional or observational studies assessing the effectiveness and safety of antiplatelet therapy in patients with aneurysmal subarachnoid hemorrhage | - Not written in English - Systematic reviews and meta-analysis, editorials, commentaries, opinion papers, letters, education papers, conference abstracts, protocols, reports, theses or book chapters - Not exclusively about subarachnoid hemorrhage (either not about subarachnoid hemorrhage at all, or about subarachnoid hemorrhage within a heterogenous population of cerebrovascular disease) - Treatment not tested in the clinical setting (e.g. lab based rather than clinical practice) - Non-human subjects (e.g. murine, porcine studies) - Not an antiplatelet - Wrong control group (e.g. control group was treated with an antiplatelet) - Did not report outcomes of interests (e.g. delayed cerebral ischemia or vasospasm) - Did not report outcome that could be extracted or disaggregated - Overlapping populations |

Supplementary Table 3. Summary of included studies

| First author, year | Country | Study design | Study period | Patients with AT, n | Patients without AT, n | Type of antiplatelet | AT dose | AT duration | Microsurgery AT, n | Endovascular AT, n | Microsurgery control, n | Endovascular control, n | Follow up |
| --- | --- | --- | --- | --- | --- | --- | --- | --- | --- | --- | --- | --- | --- |
| Al Mulfti F et al. 2021 | USA | Retrospective cohort | NR (7 years) | 18 | 168 | Aspirin and clopidogrel | NA | Pre-ictal  Reversed after aSAH | NR | NR | NR | NR | 3 months |
| Bruder M et al. 2018 | Germany | Retrospective cohort | 1999 to 2014 | 144 | 144 | Aspirin | NR | Pre-ictal  Stopped during hospital stay | 55 | 74 | 53 | 76 | 6 months |
| Darkwah Oppong M et al. 2018 | Germany | Case control | 2003 to 2016 | 329 | 251 | Aspirin and clopidogrel | Aspirin 100mg daily  Clopidogrel 75mg daily | >3 weeks  If stent applied, clopidogrel >6 weeks and aspirin use extended to lifelong. | 0 | 329 | 0 | 251 | 6 months |
| Ditz C et al. 2020 | Germany | Retrospective cohort | 2011 to 2019 | 85 | 75 | Aspirin and clopidogrel | Aspirin 300mg daily (maintenance dose 100mg)  Clopidogrel 450mg daily (maintenance dose 75mg)  Tirofiban infusion as bridge | >6 weeks | 0 | 85 | 0 | 75 | 3 months |
| Hop JW et al. 2000 | Netherlands | RCT | 1995 to 1997 | 24 | 26 | Aspirin | Aspirin 100mg suppositories | 3 weeks | 24 | 0 | 26 | 0 | 4 months |
| Juvela S et al. 1995 | Finland | Prospective cohort | NR | 62 | 144 | Aspirin | NR | Pre-ictal Stopped during hospital stay | 62 | 0 | 144 | 0 | 1 year |
| Kimura H et al. 2015 | Japan | Retrospective cohort | 2008 to 2012 | 62 | 68 | Cilostazol | Cilostazol 100mg twice daily orally | 2 weeks | 62 | 0 | 68 | 0 | Discharge |
| Matsuda N et al. 2016 | Japan | RCT | 2010 to 2013 | 74 | 74 | Cilostazol | Cilostazol 100mg twice daily orally | 2 weeks | 61 | 13 | 74 | 65 | 3 months |
| Nagahama Y et la. 2017 | USA | Retrospective cohort | 2009 to 2014 | 85 | 76 | Aspirin and clopidogrel | Aspirin 325mg daily  (maintenance dose 100mg)  Clopidogrel 600mg daily  (maintenance dose 75mg)  Tirofiban infusion as bridge | NR | 85 | 0 | 76 | 0 | 6 weeks post discharge |
| Nakatsuka Y et al. 2016 | Japan | Retrospective cohort | 2007 to 2015 | 51 | 36 | Cilostazol | Cilostazol 50mg or 100mg, twice or thrice daily orally or enterally | 2 weeks | 16 | 35 | 36 | 14 | Discharge |
| Sebok M et al. 2021 | Switzerland and the Netherlands | Prospective cohort | 2005 to 2016 | 161 | 872 | Aspirin | NR | Pre-ictal  Stopped during hospital stay | 67 | 72 | 357 | 448 | 6 months |
| Senbokuya N et al. 2013 | Japan | RCT | 2009 to 2010 | 54 | 55 | Cilostazol | Cilostazol 100mg twice daily orally | 2 weeks | 54 | 0 | 55 | 0 | 6 months |
| Sugimoto K et al. 2018 | Japan | RCT | 2013 to 2016 | 23 | 25 | Cilostazol | Cilostazol 100mg twice daily orally | 2 weeks | 23 | 0 | 25 | 0 | 3 months |
| Sun G et al. 2020 | Korea | Retrospective cohort | 2009 to 2018 | 65 | 101 | Aspirin and clopidogrel | Aspirin 100mg daily  Clopidogrel 75mg daily | NR | 0 | 65 | 0 | 101 | Discharge |
| Suzuki S et al. 1989 | Japan | RCT | 1983 to 1985 | 170 | 86 | OKY-046 | OKY-046 20 or 100 mg infusion | 10 days to 2 weeks | 170 | 0 | 86 | 0 | 3 months |
| Suzuki S et al. 2011 | Japan | RCT | 2006 to 2008 | 49 | 51 | Cilostazol | Cilostazol 100mg twice daily orally | 2 weeks | 49 | 0 | 51 | 0 | Discharge |
| Tokiyoshi K et al. 1991 | Japan | RCT | 1986 to 1989 | 13 | 11 | OKY-046 | OKY-046 1 μg/kg/min infusion | 8 days to 2 weeks | 13 | 0 | 11 | 0 | 1 month |
| Toussaint LG et al. 2004 | USA | Retrospective cohort | 1990 to 1997 | 29 | 276 | Aspirin | NR | Pre-ictal Stopped during hospital stay | 26 | 1 | 216 | 28 | Mean 16.4 months |
| van den Bergh WM et al. 2006 | Netherlands | RCT | 2000 to 2004 | 87 | 74 | Aspirin | Aspirin 100mg suppositories | 2 weeks | 53 | 33 | 55 | 19 | 3 months |
| Yano K et al. 1993 | Japan | Prospective cohort | 1989 to 1992 | 13 | 15 | OKY-046 | OKY-046 1 μg/kg/min infusion | 2 weeks | 13 | 0 | 15 | 0 | Discharge |
| Yoshimoto T et al. 2009 | Japan | Retrospective cohort | 2004 to 2006 | 26 | 24 | Cilostazol | Cilostazol 100mg twice daily orally | 2 weeks | 22 | 4 | 19 | 5 | 1 month |
| Zanaty M et al. 2020 | USA | Retrospective cohort | 2009 to 2019 | 21 | 81 | Aspirin and clopidogrel | Aspirin 325mg daily  (maintenance dose 100mg)  Clopidogrel 600mg daily  (maintenance dose 75mg)  Tirofiban infusion as bridge | NR | 0 | 21 | 81 | 0 | NR |

Supplementary Table 4. Joanna Briggs Institute quality assessment checklist for non-randomized studies

|  |  | | **Question no.** | | | | | | | | | |  | |  | |
| --- | --- | --- | --- | --- | --- | --- | --- | --- | --- | --- | --- | --- | --- | --- | --- | --- |
| **Study** | **1** | **2** | | **3** | **4** | **5** | **6** | **7** | **8** | **9** | **10** | **11** | | **Overall** | |  |
| Al Mulfti F et al. 2021 | ✔ | ✔ | | ✔ | ✔ | ✔ | ✔ | ✔ | ✔ | ✔ | ✔ | ✔ | | ✔ | |  |
| Bruder M et al. 2018 | ✔ | ✔ | | ✔ | ✔ | ✔ | ✔ | ✔ | ✔ | ✔ | ✔ | ✔ | | ✔ | |  |
| Darkwah Oppong M et al. 2018 | ✔ | ✔ | | ✔ | ✔ | ✔ | ✔ | ✔ | ✔ | ✔ | ✔ | ✔ | | ✔ | |  |
| Ditz C et al. 2020 | ✔ | ✔ | | ✔ | ✔ | ✔ | ✔ | ✔ | ✔ | ✔ | ✔ | ✔ | | ✔ | |  |
| Juvela S et al. 1995 | ✔ | ✔ | | ✔ | ✔ | ✔ | ✔ | ✔ | ✔ | ✔ | ✔ | ✔ | | ✔ | |  |
| Kimura H et al. 2015 | ✔ | ✔ | | ✔ | ✔ | ✔ | ✔ | ✔ | ✔ | ✔ | ✔ | ✔ | | ✔ | |  |
| Nagahama Y et la. 2017 | ✔ | ✔ | | ✔ | ✔ | ✔ | ✔ | ✔ | ✔ | ✔ | ✔ | ✔ | | ✔ | |  |
| Nakatsuka Y et al. 2016 | ✔ | ✔ | | ✔ | ✔ | ✔ | ✔ | ✔ | ✔ | ✔ | ✔ | ✔ | | ✔ | |  |
| Sebok M et al. 2021 | ✔ | ✔ | | ✔ | ✔ | ✔ | ✔ | ✔ | ✔ | ✔ | ✔ | ✔ | | ✔ | |  |
| Sun G et al. 2020 | ✔ | ✔ | | ✔ |  |  | ✔ | ✔ | ✔ | ✔ | ✔ | ✔ | | ✔ | |  |
| Toussant LG et al. 2004 | ✔ | ✔ | | ✔ | ✔ | ✔ | ✔ | ✔ | ✔ | ✔ | ✔ | ✔ | | ✔ | |  |
| Yano K et al. 1993 | ✔ | ✔ | | ✔ | ✔ | ✔ | ✔ | ✔ | ✔ | ✔ | ✔ | ✔ | | ✔ | |  |
| Yoshimoto T et al. 2009 | ✔ | ✔ | | ✔ | ✔ | ✔ | ✔ | ✔ | ✔ | ✔ | ✔ | ✔ | | ✔ | |  |
| Zanaty M et al. 2020 | ✔ | ✔ | | ✔ | ✔ | ✔ | ✔ | ✔ | ✔ | ✔ | ✔ |  | | ✔ | |  |

1. Were the two groups similar and recruited from the same population?

2. Were the exposures measured similarly to assign people to both exposed and unexposed groups?

3. Was the exposure measured in a valid and reliable way?

4. Were confounding factors identified?

5. Were strategies to deal with confounding factors stated?

6. Were the groups/participants free of the outcome at the start of the study (or at the moment of exposure)?

7. Were the outcomes measured in a valid and reliable way?

8. Was the follow up time reported and sufficient to be long enough for outcomes to occur?

9. Was follow up complete, and if not, were the reasons to loss to follow up described and explored

10. Were strategies to address incomplete follow up utilized?

11. Was appropriate statistical analysis used?

Supplementary Table 5. Version 2 of the Cochrane risk-of-bias assessment for randomized trials (RoB 2)

|  |  | **Domain** | | | | |  |
| --- | --- | --- | --- | --- | --- | --- | --- |
| **Study** | **Randomization process** | | **Deviations from the intended interventions** | **Missing outcome data** | **Measurement of the outcome** | **Selection of the reported result** | **Overall** |
| Hop JW et al. 2000 | Low | | Some concerns | Low | Low | Low | Some concerns |
| Matsuda N et al. 2016 | Low | | Low | Low | Low | Low | Low |
| Senbokuya N et al. 2013 | Low | | High | Low | Low | Low | High |
| Sugimoto K et al. 2018 | Low | | Low | Low | Low | Low | Low |
| Suzuki S et al. 1989 | Low | | Low | Low | Low | Low | Low |
| Suzuki S et al. 2011 | Low | | Some concerns | Low | Low | Low | Some concerns |
| Tokiyoshi K et al. 1991 | Some concerns | | High | Low | Low | Low | High |
| van den Bergh WM et al. 2006 | Low | | Low | Low | Low | Low | Low |

Some concerns

The study is judged to raise some concerns in at least one domain for this result, but not to be at high risk of bias for any domain.

High risk of bias

The study is judged to be at high risk of bias in at least one domain for this result. Or The study is judged to have some concerns for multiple domains in a way that substantially lowers confidence in the result.

Supplementary Table 6. Subgroup analyses by timing of antiplatelet administration

| Outcome | No. of studies reporting variable | Pooled effect size [95% confidence interval] | I^2^ (%) |
| --- | --- | --- | --- |
| **Post-ictal** | | | |
| Delayed cerebral ischemia | 16 | 0.52 [0.32; 0.82] | 0.0 |
| Symptomatic vasospasm | 11 | 0.56 [0.42; 0.75] | 46.3 |
| Angiographic vasospasm (moderate and severe) | 7 | 0.74 [0.64; 0.86] | 0.0 |
| Angiographic vasospasm (severe) | 6 | 0.62 [0.46; 0.84] | 0.0 |
| mRS score 0–2 | 11 | 1.18 [1.10; 1.26] | 0.0 |
| In-hospital mortality | 5 | 0.56 [0.39; 0.80] | 0.0 |
| Hemorrhagic complications | 7 | 1.36 [0.77; 2.41] | 0.0 |
| **Pre-ictal** | | | |
| Delayed cerebral ischemia | 4 | 0.96 [0.76; 1.23] | 62.3 |
| Symptomatic vasospasm | 3 | 1.24 [0.67; 2.30] | 0.0 |
| mRS score 0–2 | 3 | 0.84 [0.49; 1.43] | 74.7 |
| In-hospital mortality | 2 | 1.20 [0.01; 96.78] | 75.9 |

mRS = Modified Rankin Scale

No antiplatelet group used as control

Only outcomes with more than one included study in the subgroup analysis are reported

Supplementary Table 7. Subgroup analyses by type of antiplatelet administered

| Outcome | No. of studies reporting variable | Pooled effect size [95% confidence interval] | I^2^ (%) |
| --- | --- | --- | --- |
| **Cilostazol** | | | |
| Delayed cerebral ischemia | 7 | 0.40 [0.32; 0.49] | 0.0 |
| Symptomatic vasospasm | 6 | 0.47 [0.33; 0.65] | 0.0 |
| Angiographic vasospasm (moderate and severe) | 5 | 0.75 [0.57; 0.98] | 20.3 |
| Angiographic vasospasm (severe) | 4 | 0.59 [0.28; 1.27] | 27.9 |
| mRS score 0–2 | 7 | 1.24 [1.08; 1.43] | 24.2 |
| In-hospital mortality | 2 | 0.23 [0.00; 34.81] | 0.0 |
| Hemorrhagic complications | 2 | 0.64 [0.00; 1306991.97] | 45.6 |
| **Aspirin and clopidogrel** | | | |
| Delayed cerebral ischemia | 5 | 0.40 [0.10; 1.53] | 70.5 |
| Symptomatic vasospasm | 5 | 0.59 [0.26; 1.36] | 57.1 |
| Angiographic vasospasm (moderate and severe) | 2 | 0.76 [0.59; 0.97] | 0.0 |
| mRS score 0–2 | 2 | 1.15 [0.80; 1.65] | 0.0 |
| In-hospital mortality | 2 | 0.60 [0.41; 0.88] | 0.0 |
| Hemorrhagic complications | 4 | 1.46 [0.61; 3.46] | 4.3 |
| **Aspirin** | | | |
| Delayed cerebral ischemia | 6 | 0.91 [0.64; 1.30] | 64.3 |
| Symptomatic vasospasm | 2 | 1.27 [1.11; 1.45] | 0.0 |
| mRS score 0–2 | 4 | 0.90 [0.61; 1.34] | 75.0 |
| In-hospital mortality | 2 | 1.20 [0.01; 96.78] | 75.9 |

mRS = Modified Rankin Scale

No antiplatelet group used as control

Only outcomes with more than one included study in the subgroup analysis are reported

Supplementary Table 8. Subgroup analyses by treatment modality

| Outcome | No. of studies reporting variable | Pooled effect size [95% confidence interval] | I^2^ (%) |
| --- | --- | --- | --- |
| **Microsurgery** | | | |
| Delayed cerebral ischemia | 8 | 0.58 [0.26; 1.31] | 72.7 |
| Symptomatic vasospasm | 6 | 0.55 [0.30; 0.98] | 71.0 |
| Angiographic vasospasm (moderate and severe) | 4 | 0.70 [0.54; 0.90] | 0.0 |
| Angiographic vasospasm (severe) | 3 | 0.61 [0.21; 1.81] | 24.1 |
| mRS score 0–2 | 7 | 1.23 [1.09; 1.41] | 12.5 |
| In-hospital mortality | 4 | 0.65 [0.28; 1.52] | 0.0 |
| Hemorrhagic complications | 2 | 1.30 [0.15; 11.50] | 0.0 |
| **Endovascular** | | | |
| Delayed cerebral ischemia | 5 | 0.37 [0.11; 1.21] | 65.8 |
| Symptomatic vasospasm | 4 | 0.60 [0.20; 1.80] | 64.5 |
| mRS score 0–2 | 2 | 1.15 [0.80; 1.65] | 0.0 |
| In-hospital mortality | 2 | 0.60 [0.41; 0.88] | 0.0 |
| Hemorrhagic complications | 4 | 1.46 [0.61; 3.46] | 4.3 |

mRS = Modified Rankin Scale

No antiplatelet group used as control

Only outcomes with more than one included study in the subgroup analysis are reported

Supplementary Table 9. Sensitivity analysis of only post-ictal studies with subgroup analysis by treatment modality

| Outcome | No. of studies reporting variable | Pooled effect size [95% confidence interval] | I^2^ (%) |
| --- | --- | --- | --- |
| **Microsurgery** | | | |
| Delayed cerebral ischemia | 7 | 0.51 [0.20; 1.32] | 68.0 |
| Symptomatic vasospasm | 5 | 0.44 [0.30; 0.64] | 0.0 |
| Angiographic vasospasm (moderate and severe) | 4 | 0.70 [0.54; 0.90] | 0.0 |
| Angiographic vasospasm (severe) | 3 | 0.61 [0.21; 1.81] | 24.1 |
| mRS score 0–2 | 7 | 1.23 [1.09; 1.41] | 12.5 |
| In-hospital mortality | 3 | 0.27 [0.10; 1.04] | 0.0 |
| Hemorrhagic complications | 2 | 1.30 [0.15; 11.50] | 0.0 |
| **Endovascular** | | | |
| Delayed cerebral ischemia | 5 | 0.37 [0.11; 1.21] | 65.8 |
| Symptomatic vasospasm | 4 | 0.60 [0.20; 1.80] | 64.5 |
| mRS score 0–2 | 2 | 1.15 [0.80; 1.65] | 0.0 |
| In-hospital mortality | 2 | 0.60 [0.41; 0.88] | 0.0 |
| Hemorrhagic complications | 4 | 1.46 [0.61; 3.46] | 4.3 |

mRS = Modified Rankin Scale

No antiplatelet group used as control

Only outcomes with more than one included study in the subgroup analysis are reported

Supplementary Figure 1. PRISMA flow diagram for studies included and excluded from the systematic review and meta-analysis.

Figure 2. Forest plots with random-effects model, stratified by treatment modality, of A) delayed cerebral ischemia, B) symptomatic vasospasm, C) moderate/severe angiographic vasospasm, D) good functional outcome (mRS0-2), E), in-hospital mortality, F), hemorrhagic complications
